# The Influence of New and Artificial Aged Microplastic and Leachates on the Germination of *Lepidium* *sativum* L.

**DOI:** 10.3390/plants9030339

**Published:** 2020-03-07

**Authors:** Stephan Pflugmacher, Amalia Sulek, Hannah Mader, Jeongin Heo, Ji Hyeon Noh, Olli-Pekka Penttinen, YoungJun Kim, Sanghun Kim, Maranda Esterhuizen

**Affiliations:** 1 Aquatic Ecotoxicology in an Urban Environment, Faculty of Biological and Environmental Sciences, Ecosystems and Environment Research Programme, University of Helsinki, Niemenkatu 73, 15140 Lahti, Finland; amaliasophie@hotmail.com (A.S.); hannah.mader95@googlemail.com (H.M.); sgjwjddls12@gmail.com (J.H.); njh2535@gmail.com (J.H.N.); olli-pekka.penttinen@helsinki.fi (O.-P.P.); 2 Joint Laboratory of Applied Ecotoxicology, Environmental Safety Group, Korea Institute of Science and Technology Europe (KIST Europe) Forschungsgesellschaft mbH, Universität des Saarlandes Campus E7 1, 66123 Saarbrücken, Germany; youngjunkim@kist-europe.de; 3 Helsinki Institute of Sustainability Science (HELSUS), University of Helsinki, Fabianinkatu 33, 00014 Helsinki, Finland; 4 Department of Pharmaceutical Science and Technology, Centre for Chemical Safety Research, Kyungsung University, 309, Suyeong-ro, Nam-gu, Busan 48434, Korea; fatherofdamin@ks.ac.kr

**Keywords:** polycarbonate, microplastic, artificially aged microplastic, leached microplastic, leaching solution, bisphenol A, seed germination parameters, *Lepidium sativum*

## Abstract

With the increase in environmental monitoring and assessing, we are gaining insight into the extent of microplastic pollution in our environment. The threat posed by microplastics to biota could come, e.g., from leached substances. As some plastic materials have been decaying in nature for extended periods already, the toxic effects of leaching compounds need to be investigated. It is furthermore essential to understand the adverse effects of new plastic and how these effects differ from the effects elicited by old plastic material. Therefore, in the present study, the effects of exposure to leachates from new and artificial aged polycarbonate as well as new and aged polycarbonate granules on various germination parameters of *Lepidium sativum* were studied. Germination, root, and shoot length, as well as the calculated germination rate index as a measure for germination speed, was negatively influenced in substrate-free and substrate containing exposures. From an ecological and agricultural point of view, this implies possible yield losses with less germinating seeds, slower plant germination speed, and smaller seedlings in general.

## 1. Introduction

Most human activities nowadays are based on or influenced by plastics and plastic products. Plastic is essential in nearly all aspects of human life. Due to intensive production, around 8.3 million metric tons of virgin plastic were produced in 2015 [1]. Due to its ubiquitous use, low cost, and the single-use throwaway culture, plastic pollution has become a severe environmental problem [2]. Plastic debris as a source of waste and chemical pollution is detected not only in oceans, forming huge swirling patches in areas, but also soil, sediment, freshwater bodies, and even human stool [3,4].

The threats posed by microplastics (MP) in nature need to be investigated. However, a standardized definition of what can be defined as MPs in terms of size classification is still under debate, and numerous definitions can be found. The European Marine Strategy Framework Directive Working Group on Good Environmental Status defines the following classes: macroplastics (> 25 mm), mesoplastics (5 to 25 mm), large microplastics (1 to 5 mm), and small microplastics (< 1 mm) [5]. It is apparent that plastics will degrade into MP and that MP particles are not static in size and will be further degraded into much smaller particles with time due to environmental impacts [6], making uptake in organisms and even crossing membranes possible [7]. MPs introduced into our environment could be primary MPs in specific applications or secondary MPs, as a result of larger plastic parts decaying due to environmental actions (wind, UV-light, water, ice). Around 110,000 to 730,000 tons of MPs are annually transferred to agricultural soils in Europe and North America, which is more than the total burden of MPs currently in our oceans [8,9,10]. In a study on macro- and microplastics in agricultural soil, up to 205 macroplastic pieces per hectare (ha) and 0.34 ± 0.36 MPs pieces per kilogram soil (dry weight) were detected; illustrating the vast extent of soil contamination [11]. Entry routes of MPs into terrestrial systems are not well known; however, initial studies have attempted to elucidate these possible pathways, such as, for example, via the use of organic fertilizers as a vehicle for MP entry [12,13].

Terrestrial ecosystems might face MPs pollution earlier than the aquatic ecosystems, and it is currently unknown what impacts MPs might have on the exposed organisms in soil or other substrate systems. Changes in soil structure and terrestrial geochemistry (water holding capacity, hydraulic conductivity, soil aggregation and microbial activity) already have been shown [14,15] which could affect the soil community [16].

Initially, plastic polymers were thought to be inert and therefore of low concern in terms of toxicity and thus pose no adverse effects to consumers or the environment. Plastics contain additives [17,18] which provide particular characteristics to the product such as, e.g., bio-stabilizers, antimicrobials, antioxidants, antistatic agents, blowing agents, fillers/extenders, flame retardants, fragrances, heat stabilizers, light stabilizers, pigments, and process aids [18]. These additives could, however, leach out into the surrounding environment and accumulate in water, sediment, food, or even body tissues [19]. The effects of MPs as a whole, chemicals that leach from them, as well as surface-bound chemicals on biota, are still unclear and due to the presence of MP in substrates, the possible impacts on agriculture and thus our primary source of sustenance such as food crops need further investigation.

The most prominent plastic types are polyethylene (PE), polypropylene (PP), polycarbonate (PC) polyvinylchloride (PVC), polyurethane (PUR), PE terephthalate (PET), and polystyrene (PS). Approximately one million tons of PC are produced annually. PCs, which are thermoplastic polymers containing carbonate groups, have a variety of applications including glass applications for lenses and windows, flame retardant, electrical insulator, compact disks (CDs) and digital video disks (DVDs), automotive industry, cell phones, and laptop parts. PCs are produced via a step-growth polymerization (condensation process) using bisphenol A (BPA) and phosgene (COCl_2_) to eliminate Cl-ions [20]. Usually, BPA is not released from the plastic matrix unless the plastic undergoes degradation. Therefore, based on the high usage and chemical properties of PC, it was selected as a model plastic to investigate.

In the present study, we investigated not only the effects of new PC MP but also artificially aged PC MP, leached PC MP, the leached solutions, and one of the building blocks of PC, bisphenol A, to get a better insight regarding how MPs would influence a crop plant in soil. We address the possible effects of a substrate, performing all experiments with garden cress (*Lepidium sativum)* seeds in substrate-free and in a substrate-containing environment, in parallel. The aim of the present study was therefore to understand the effects of new, artificially aged, and leachates from new and old PC on the germination of *L. sativum* in a substrate and substrate-free system as germination is when plants are at their most vulnerable to environmental stresses and imbibe the most water, i.e., would be exposed to MP leachates.

## 2. Results and Discussion

Plant seed coats create a natural barrier between a plant embryo and the surrounding environment to protect against contamination until the radicle starts to develop. The overall germination process constitutes three principal processes: the imbibition, the activation, and the intra-seminal growth, leading to the embryo protrusion [21]. During germination, the plant is most vulnerable to dissolved toxic substances because the seeds imbibe plenty of water from its surroundings through pores in the seed capsule [22]. Therefore, the effects, primarily on germination, was considered as leached substances in water could affect the plants (leachates) or MP particles could alter soil properties affecting water availability (granules).

### 2.1. Germination in Substrate-Free System with Leachates

**Figure 1:**
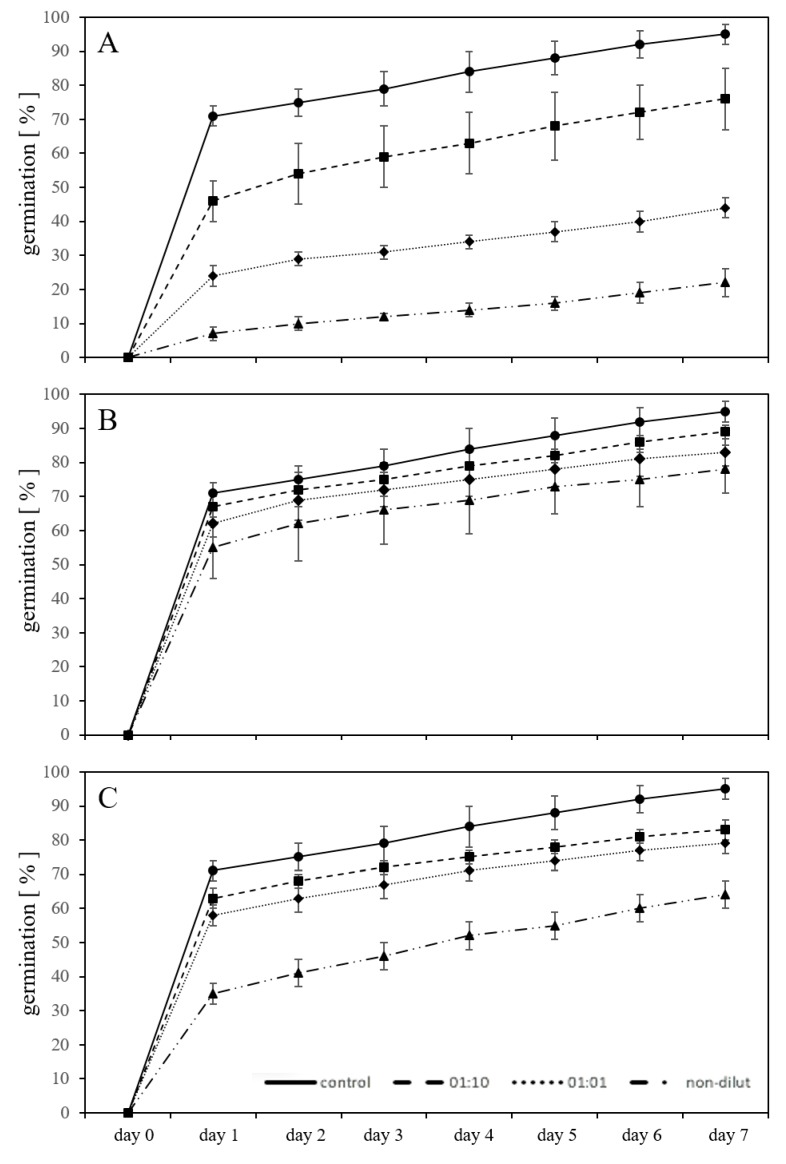
Germination percentage of *L. sativum* seeds exposed in a substrate-free environment to leachates of new (**A**) and artificially aged (**B**) polycarbonate microplastics as well as three different concentrations of bisphenol A (**C**) for seven days, showing adverse effects in the form of inhibition of germination compared to untreated controls.

Using germination as a test parameter in substrate-free exposures, the leachate solution from the new PC material (*new-PC-leachate*), in the two dilutions 1:10 and 1:1, as well as non-diluted, significantly inhibited the germination of *L. sativum* compared to the control (*p* < 0.05) (Figure 1A). After seven days of exposure, the germination was inhibited by 20% in the 1:10 diluted leachate, 54% in the 1:1 diluted leachate, and by 77% using the non-diluted leachate compared to untreated controls. A strong inhibitory effect compared to untreated controls was seen using the artificially aged PC leachate (*aged-PC-leachate*) (*p* < 0.05), for which the germination was inhibited by 6% using 1:10 diluted leachate, 13% using 1:1 diluted leachate, and 18% using the non-diluted leachate. (Figure 1B). Comparing the inhibitory effect on germination in the presence of the *new-PC-leachate* with the *aged-PC-leachate,* it is evident that ageing decreased the ecotoxicity of the plastic material. For example, the non-diluted *aged-PC-leachate* inhibited the germination less than the 1:10 diluted *new-PC-leachate*. Exposure of the seeds to three different concentrations of BPA (10, 50, and 100 mg/L), a primary component of PC and also a known endocrine-disrupting chemical [23], exhibited inhibitory effects on the germination after seven days. The inhibition with exposure to 10 mg/L BPA was 13%, using 50 mg/L BPA 17% inhibition, and using 100 mg/L 33% inhibition was observed (Figure 1C). The degree of inhibition on the germination by BPA falls between the results of the *new-PC-leachate* and the *aged-PC-leachate,* suggesting that the adverse effects resulting from the *new-PC-leachate* are the sum of numerous harmful substances leaching from the PC. Dogan et al. [24] reported that BPA at concentrations of 10 mg/L and 50 mg/L did not cause inhibited germination of chickpea (*Cicer arietinum* L. cv. Ispanyol); however, a BPA concentration of 50 mg/L was able to reduce the germination of *Triticum aestivum* L. cv. Sagittario significantly by 43% in a substrate-free system [25]. It is possible that like *T. aestivum*, *L. sativum* is more sensitive to BPA exposure than *C. arietinum*. Exposure of *Lens culinaris* seeds to 10, 50, and 100 mg/L BPA in a substrate-free system also caused inhibited germination [26]. These observed discrepancies illustrate that different plants have different sensitivities to the effects of MP, which is a theory previously suggested by Rillig et al. [27].

### 2.2. Germination in a Substrate-System with Exposure to Leachates

**Figure 2:**
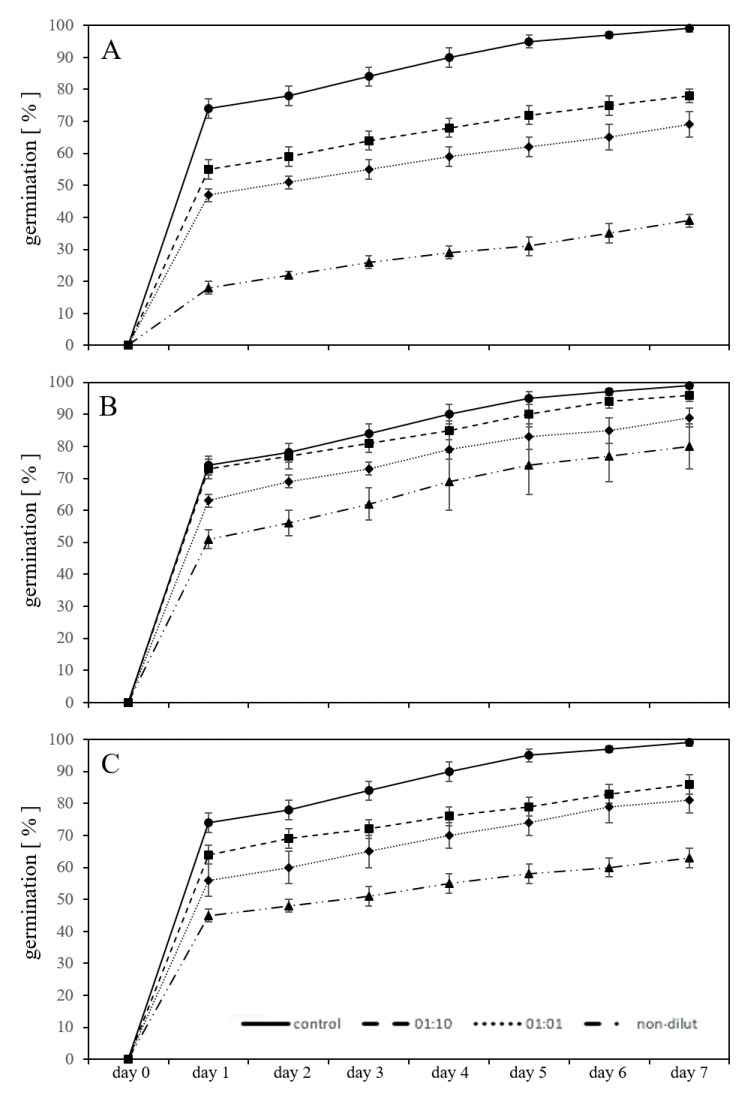
Adverse effect on germination percentage of *L. sativum* seeds exposed to leachates of new (**A**), artificially aged (**B**) polycarbonate microplastic particles and bisphenol A (**C**) in the substrate for seven days compared to untreated controls in the substrate.

The same experimental set-up detailed above was repeated in a substrate. The *new-PC-leachate* in different dilutions (1:10, 1:1, and non-diluted) exhibit a lower, but still significant (*p* < 0.05) inhibition of germination. After seven days, using the 1:10 diluted leachate, the germination of *L. sativum* was inhibited by 21% compared to the controls. The inhibition was 30% in comparison to untreated controls using the 1:1 diluted leachate and 61% using the non-diluted leachate (Figure 2A). With *aged-PC-leachate,* the inhibition of the germination from untreated controls after seven days was 3% using the 1:10 diluted leachate, 10% using the 1:1 diluted leachate, and 19% with the non-diluted leachate (Figure 2B). By ageing the plastic, the inhibitory effect of the used plastic leachate was diminished by one third. Exposures with the three concentrations of BPA in substrate revealed significant inhibition of the germination (*p* < 0.05) by 13% with exposure to 10 mg/L, by 18% using 50 mg/L and by 36% 100 mg/L from untreated controls (Figure 2C). In addition, in exposures with the leachates and BPA in the substrate systems, the severity of the germination inhibition caused by BPA fell between the values of *new-PC-leachate* and *aged-PC-leachate*.

The buffer function of the substrate is evident for exposures to the *new-PC*- and *aged-PC*-leachate showing in most cases slightly lower germination inhibitions. Yet, in exposures using BPA, the inhibitory effect in both systems was nearly the same. However, Rillig et al. [27] pointed out that in nature, the natural microbiota may be affected by the presence of plastics in soil and thus the toxic substances associated with and leaching from MP. Therefore, even though the substrate system seemed to buffer the effects of the leachates, the combined effects of both MP and the leachates should be taken into account to understand the entirety of the effects on plants.

Polycyclic aromatic hydrocarbons (PAHs) are commonly detected in soils as xenobiotics and was previously found to inhibit the germination of *L. sativum* by > 84% and < 25%, at exposure concentrations of 1000 and 50 mg/kg, respectively [28]. Relating this to the results found in the present study, exposure to non-diluted PC leachate roughly elicits the same effect as exposure to between 50 and 1000 mg/kg PAHs.

### 2.3. Germination in a Substrate-System with MP Granules

**Figure 3:**
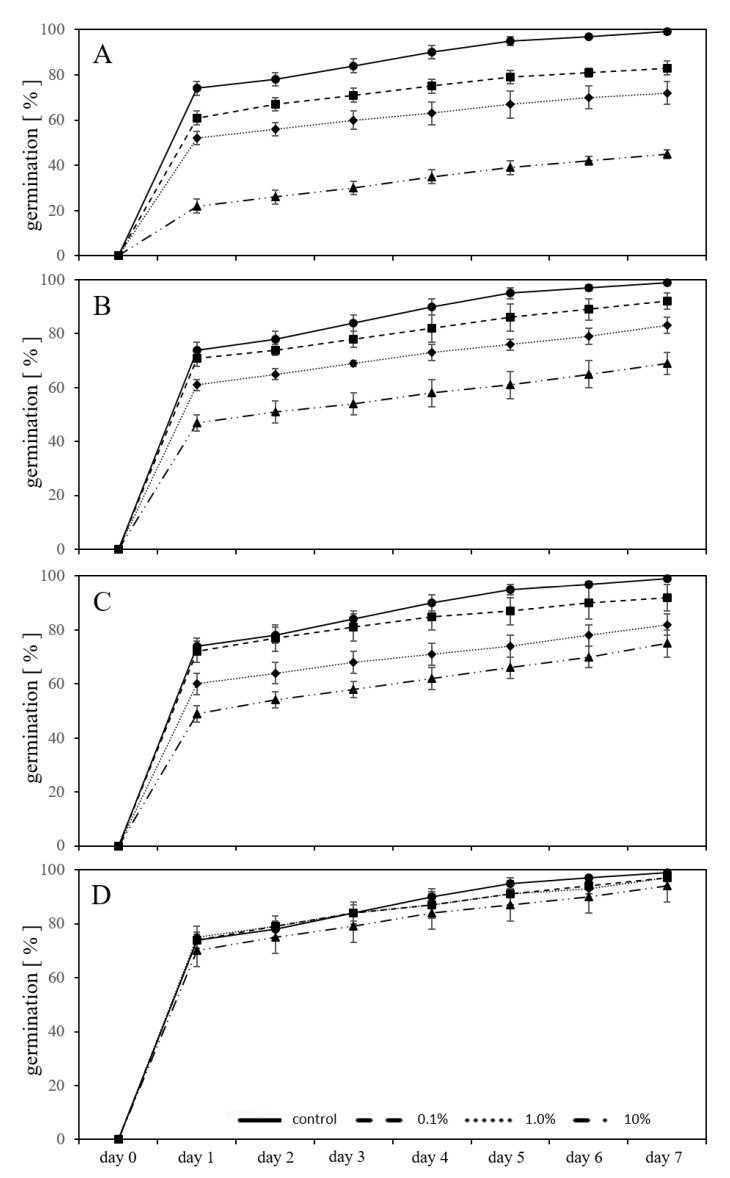
Adverse effects on germination percentage of *L. sativum* seeds exposed to various concentrations of *new-PC-granules* (**A**), *aged-PC-granules* (**B**), pre-*leached new-PC-granules* (**C**) and pre-*leached-aged-PC-granules* (**D**) during seven days of germination in a substrate with exposure, compared to untreated controls.

The inhibitory effect on the germination of *L. sativum* was also measured in exposures using *new-PC-granules* without prior leaching, mixed in the substrate in three different percentages (w/w) (Figure 3A). The germination was inhibited (*p* < 0.05) from the first day. After seven days of exposure to 0.1% of *new-PC-granules* in the substrate, the inhibition of the germination was 16%, in substrate containing 1% *new-PC-granules* it was 27%, and 55% in substrate containing 10% of *new-PC-granules* compared to untreated controls.

Using *aged-PC-granules* without prior leaching, mixed in the substrate in three different percentages (w/w) (Figure 3B), the germination was inhibited (*p* < 0.05) from the first day. However, with exposure to 0.1% *aged-PCgranules* mixed in the substrate, the germination was not significantly influenced compared to the control from day 1 to 4 (*p* > 0.05).

Comparing the inhibitory effect of *new-PC-granules* and *aged-PC-granules*, the inhibition was lowered by 44% (0.1% granules in substrate), 59%, (1% granules in substrate), and 56% (10% granules in substrate), respectively, using the aged granules, clearly showing that ageing reduced the adverse effect on the germination of *L. sativum* in all granule percentages used in substrate.

Previously, Bosker et al. [29], found that MP particles (10^3^–10^7^ particles/mL) in a substrate-free system caused significant inhibition, up to 83% after 8 h, on *L. sativum* germination; however, the seeds recovered after 24 h resulting in up to 100% germination. The inhibition was said to be due to a physical blockage of the pores in the seed capsule. In the present study, the inhibition was constant over a period of seven days, and the seeds did not recover as in the case of Bosker et al. [29]. However, their experimental system was conducted in a soil-free system. It is possible that the soil wrapped the seed and MP together with no movement and therefore the MP continued to block the pores. Wan et al. [30] reported that plastics could lead to the formation of channels in the soil, which could, in turn, lead to faster drying. This could have a negative impact not only on the water availability for germinating seed but also on the soil microbe composition [30] and may have been another factor contributing to the continued inhibition seen in the present study.

The same setup using previously leached granules from *new-PC* and *aged-PC* showed in the cases of the leached *new-PC-granules* (*p* < 0.05) an inhibition of germination of 7% growing seedlings in 0.1% plastic containing substrate, 17% and 24% inhibition of germination in substrate containing 1% and 10% plastic granules compared to untreated controls (Figure 3C). Using leached *aged-PC-granules,* the inhibition was not significant compared to untreated controls and ranged between 2% (with 0.1 and 1% plastic in the substrate) and 5% (with 10% plastic in the substrate) (Figure 3D). Comparing the inhibitory effect on germination between *new-PC-granules* leached and *aged-PC-granules* leached, the inhibitory effect was lowered by 29% (0.1% granules in the substrate), 12% (1% granules in the substrate), and 21% (10% granules in the substrate) using the aged leached granules. This again supports the previous observation that ageing reduced the adverse effect on germination of *L. sativum* but also that leaching prior to exposure reduced the adverse effect.

Leaching per se in all granule percentages used in the substrate from *new-PC-granules* showed a reduced inhibitory potential from leached granules by 63% (in 0.1% granules in soil), 20% (in 1% granules in the substrate), and 38% (10% granules in the substrate). There is a variation in the reduced inhibitory effect; however, clearly, pre-leaching reduced the adverse effect of the plastic particles. Considering the *aged-PC-granules* and comparing the leached granules versus the non-leached granules, it indicates that the inhibitory effect was reduced by 29% (0.1% granules in the substrate), 13% (1% granules in the substrate), and 17% (10% granules in the substrate). In correspondence with the previously attained results, leaching of *aged-PC-granules* is reducing the adverse effect. This suggests that the compounds leaching from MP plays a significant role in the effects seen on germination.

### 2.4. Root and Seedling Length in Substrate-Free Exposures

**Figure 4:**
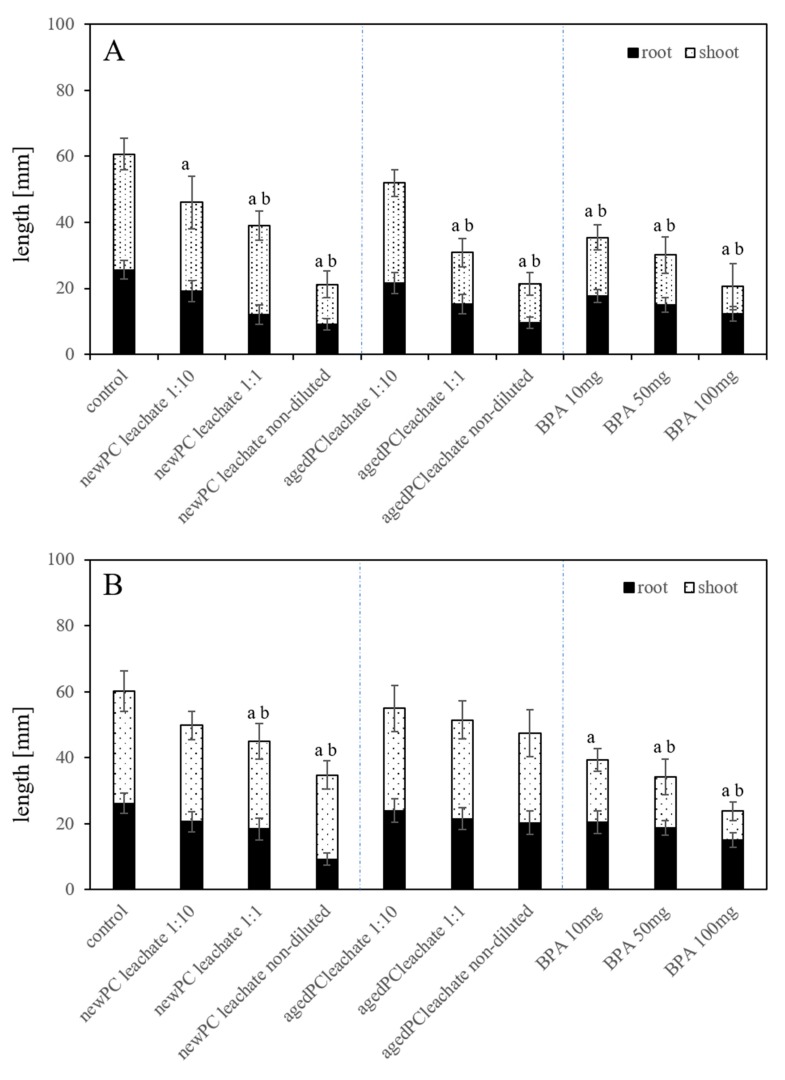
Average root length and seedlings length of *L. sativum* exposed to leaches in different dilutions from (**A**) *new-PC* and (**B**) artificially *aged-PC* as well as three different concentrations of BPA in a substrate-free environment ± standard deviation (*n* = 50). Significance (*p* < 0.05) is presented with a) for root data and b) for whole seedling length compared to the controls.

Measurement of the root and seedling length, as sub-lethal endpoints, in substrate-free exposures resulted in a concentration-dependent inhibition using the *new-PC-leachate*, the *aged-PC-leachate,* and BPA (Figure 4A). In general, the root length was reduced in all exposures (*p* < 0.05). The root growth reduction is concentration-dependent with leachates originating from *new-PC* and *aged-PC*, as well as using different BPA concentrations. Using the non-diluted *new-PC-leachate,* the reduction compared to the untreated controls is 64%. Similarly, with the non-diluted *aged-PC-leachate,* the root growth was inhibited by 63%. The highest reduction was observed in exposures with non-diluted leachate and 100 mg/L BPA (*p* < 0.05). The inhibition of root growth in seedlings exposed to BPA in high concentrations (17.2 or 50 mg/L) was also detected in soybean, wheat, and lettuce after seven days of growth [31,32]. The most provoked effect was measured in exposures with 100 mg/L BPA showing 52% smaller seedlings, compared to untreated controls. In soybean seedlings, the root length was reduced when exposed to 17.2 and 50 mg/L BPA [31]. The seedling length was adversely affected in all treatments (*p* < 0.05) in respect to untreated controls, which was concentration-dependent using *new-PC-leachate*, *aged-PC-leachate,* as well as in the BPA exposures. Using the *new-PC-leachate,* growth was reduced by 23% using the 1:10 and the 1:1 dilutions of the leachate and by 66% with the non-diluted leachate compared to untreated controls. With *age-PC-leachate,* the inhibition of seedling length was 13%, 56%, and 67% for exposure to the dilutions 1:10, 1:1 and non-diluted leachate in relation to untreated controls. Exposures to BPA showed a reduced seedling length of 50%, 57%, and 77% compared to the untreated control seedlings. Results indicated that there are adverse effects on root and total seedlings growth under the influence of plastic leachates. Especially with a higher leachate concentration (non-diluted), the adverse effects on smaller length was mostly over 66% in respect to untreated controls. As stated by Nicola [33], the root architecture is most vital for plant productivity. Therefore, the good growth of the primary root length of seedlings may determine the optimum root systems and influence the entire life cycle of this plant. For agriculturally used plants, this may also determine the later yield of this plant [34].

### 2.5. Root and Seedling Length in Substrate Exposures

In substrate systems, with exposure to *new-PC-leachate*, *aged-PC-leachate,* and BPA, a similar effect on the growth inhibition on primary root length and the seedling length was visible compared to untreated controls (Figure 4B). Root length was reduced using *new-PC-leachate* with 1:10, 1:1 diluted leachate, and non-diluted leachate. The growth inhibition was significant only in 1:1 and non-diluted exposures showing 71% and 76% reduction from untreated controls (*p* < 0.05). Using the *aged-PC-leachate* the growth inhibition in respect to untreated controls were 20% (1:10 diluted leachate), 28% (1:1 diluted leachate), and 49% (non-diluted leachate) respectively (Figure 4B). Exposure to BPA in the substrate system showed a reduced root length of 42% using 10 mg/L BPA, 55% with 50 mg/L BPF, and 74% with 100 mg/L BPA compared to untreated controls (*p* < 0.05) (Figure 4B). For *T. aestivum* variant Sagittario, BPA decreased the root length by 19.6% at an exposure concentration of 50 mg/L [25].

The leachates of *new-PC* and *aged-PC* concentration-dependently reduced the seedling length. Using *new-PC-leachate* the reductions were 14% (1:10 diluted leachate), 24% (1:1 diluted leachate), and 46% (non-diluted leachate) (*p* < 0.05) compared to the untreated control seedlings (Figure 4B). Lesser reductions concerning seedling length was seen using *aged-PC-leachate;* i.e., by 8% (1:10 diluted leachate), 17% (1:1 diluted leachate), and 22% (non-diluted leachate) (*p* < 0.05). Exposure to BPA showed a reduced seedling length of 4% using 10 mg/L BPA and 14% using both 50 mg/L and 100 mg/L BPA (*p* < 0.05) (Figure 4B). A decrease in shoot length was observed in exposures using 50 mg/L BPA with 26.6% in *T. aestivum* [25]. This adverse effect might be explained by the possible inhibition of cell elongation and cell division, as suggested by Ferrara et al. [31].

**Figure 5:**
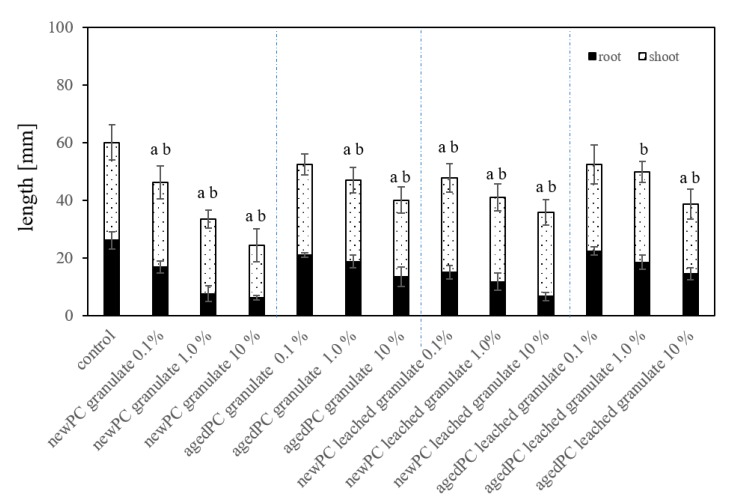
Average root length and seedlings length of *L. sativum* exposed to granules of *new-PC* and *aged-PC* mixed in the substrate in three different concentrations (w/w), as well as already leached granules from *new-PC* and *age-PC, ±* standard deviation (*n* = 50). Significance (*p* < 0.05) is represented by a) for root length and b) for seedling length.

Testing the different PC granules, *new-PC*, *aged-PC,* and already *leached new-PC* and *aged-PC*-*granules* in substrate system exposures (Figure 5) exhibited inhibitory effects on root length and total seedling length. Compared to the untreated controls, exposure to the *new-PC-granules* mixed in substrate resulted in the root growth being inhibited by 35% (0.1% *new-PC-granules* mixed in the substrate), 71%, and 76% (1% and 10% *new-PC-granules* mixed in the substrate) (*p* < 0.05). Artificially *aged-PC-granules* exhibit a lesser inhibition of 8% (0.1% *aged-PC-granules* mixed in substrate) and 17% as well as 22% (1% and 10% *aged-PC-granules* mixed in substrate) (*p* < 0.05). The same experimental set up using previously leached granules from *new-PC* and *aged-PC* batches revealed a root growth inhibition of 4%, 14%, and 14% respectively. Rillig et al. [27] proposed that MP in soil could lead to reduced roots penetration resistance, as well as better soil aeration, which would promote root growth [35]. However, in the present study, this was not the case, presumably because the effects of substances leaching from the MP was greater than the added benefits of MP.

### 2.6. Germination Rate Index Calculation

The germination rate index (GRI) expresses the percentage of germination on each day during the seven days germination periods (Table 1). Higher GRI values indicate higher and faster germination of the seeds [36]. Compared to controls exhibiting a GRI of 1.59 in substrate-free exposures, exposures to *new-PC-leachate* and *aged-PC-leachate*, as well as in exposures with BPA resulted in a reduced GRI (*p* < 0.05) and indicated lower and slower germination of the seeds in the respective treatments. Using the *new-PC-leachate*, this inhibition was 23%, 61%, and 86% for the three dilutions 1:10, 1:1, and undiluted respectively, compared to untreated controls. Whereas the *aged-PC-leachate* exhibit a GRI lowered by 6%, 14%, and 30% using the different leachate concentrations (*p* < 0.05). Exposure to the three different BPA concentrations revealed GRI’s lowered by 8%, 16%, and 42% compared to controls (*p* < 0.05). In the substrate-free exposures, the most substantial adverse effect was seen with *new-PC-leachate*, followed by BPA exposure followed by *aged-PC-leachate*.

Calculations of GRI in exposures using the substrate-system revealed, in general, using the *new-PC-leachate* resulted in a GRI lowered by 25%, 34%, and 71% compared to the control (*p* < 0.05). Using the *aged-PC-leachate,* the GRI has reduced with exposure to the 1:1 diluted leachate by 26% and with non-diluted leachate by 39%. Compared to the *new-PC-leachate*, this shows that the *aged-PC-leachate* has a reduced potential to influence the germination speed of the seeds adversely. In substrate, exposure to BPA exhibited a feeble inhibitory effect significant in treatments using 50 mg/L (13%) and 100 mg/L (24%) BPA compared to untreated controls (*p* < 0.05).

Treatments using the PC granules exhibited a lower GRI using the *new-PC-granules* mixed in the substrate in three percentages by 15%, 30%, and 65% from untreated controls (*p* < 0.05). As seen with the *aged-PC-leachate*, also the *aged-PC granules* showed a weaker influence on the GRI as it was reduced by 6%, 20%, and 35%. Using already leached *new-PC granules*, the reduction of GRI compared to untreated controls was nearly halved to 8%, 19%, and 34% also compared to the results from the respectively used leachate samples. Already *leached aged-PC* granules showed a non-significant GRI reduction by 1%, 2%, and 8% from untreated controls.

In most of the treatments, the GRI and therefore the germination speed of the *L. sativum* seeds were reduced when exposed to leachates or PC granules material. A possible ecological implication is that crop yield might be reduced if the agricultural soil contains too many plastic particles, which are able to influence the GRI, by delayed growth and seed production.

In soil, fertilizers made from recycled waste, as well as street runoff, including abrasions from tires and breaks may be a source of MP [37]. The presence of MP could change soil structure and behavior [14,15]; nevertheless, the main effect seems to be the chemicals leaching out of the plastic material.

## 3. Materials and Methods

### 3.1. Lepidium sativum (L.) Seeds

Garden cress (*Lepidium sativum* L.) seeds (Art. No. G250) were purchased from Bingenheimer Saatgut AG (Echzell, Germany). Seeds were carefully selected by removing small seeds or any deformed or damaged seeds. The seeds were then washed in distilled water to remove possible impurities. The batch used for all experiments exhibited a germination percentage of 99%. Germination experiments were performed at 25 °C ± 1 °C under 1500 lux light and a light-dark cycle of 12:12 h for seven days in total.

### 3.2. Microplastics, Aged Microplastics and Plastic Leachates

For the experiments, commercially available colorless polycarbonate granules (PC) (Goodfellow GmbH, Hamburg, Germany) with an average size of 3 mm ± 1 mm were used. The plastic material was washed with ISO reconstituted water (pH 7.2) [38] and dried at 25 °C before use to remove any superfine particles.

Accelerated ageing of the PC material was done according to Fejdyś et al. [39] making use of PN-EN 12280-1:2002. In short, artificial ageing was done in a thermal chamber (TK 720 Binder GmbH, Tuttlingen, Germany) with a heating effect at a temperature of 70 °C ± 0.5 °C and < 1.5% humidity for 80 days.

The PC was subjected to treatment to induce leaching according to the Swedish standard 12457: 2003 [40], with some modifications. Leaching time was increased from 24 h to 72 h and a temperature of 50 °C was used to increase leaching and to simulate a more realistic scenario; i.e., that the surface of plastic material in the environment may reach this temperature and even above when exposed to sunlight outdoors [41]. The leaching was accomplished by mixing 200 g of the PC material with ISO reconstituted water (pH 7.2) [38] to reach a liquid-to-solid ratio (L/S) of 10. The complete leaching process was done in darkness in round bottom flasks on a rotary mixer (Hei-VAP Valve, Heidolph-Instruments, Schwabach, Germany) at 21 rpm. The liquid and solids were separated by vacuum filtration on Whatman borosilicate glass microfiber filters (grade GF/F; particle retention 0.7 μm). This filter is approved in the EPA method TCLP 1311 for toxicity characteristic leaching procedure.

**Figure 6:**
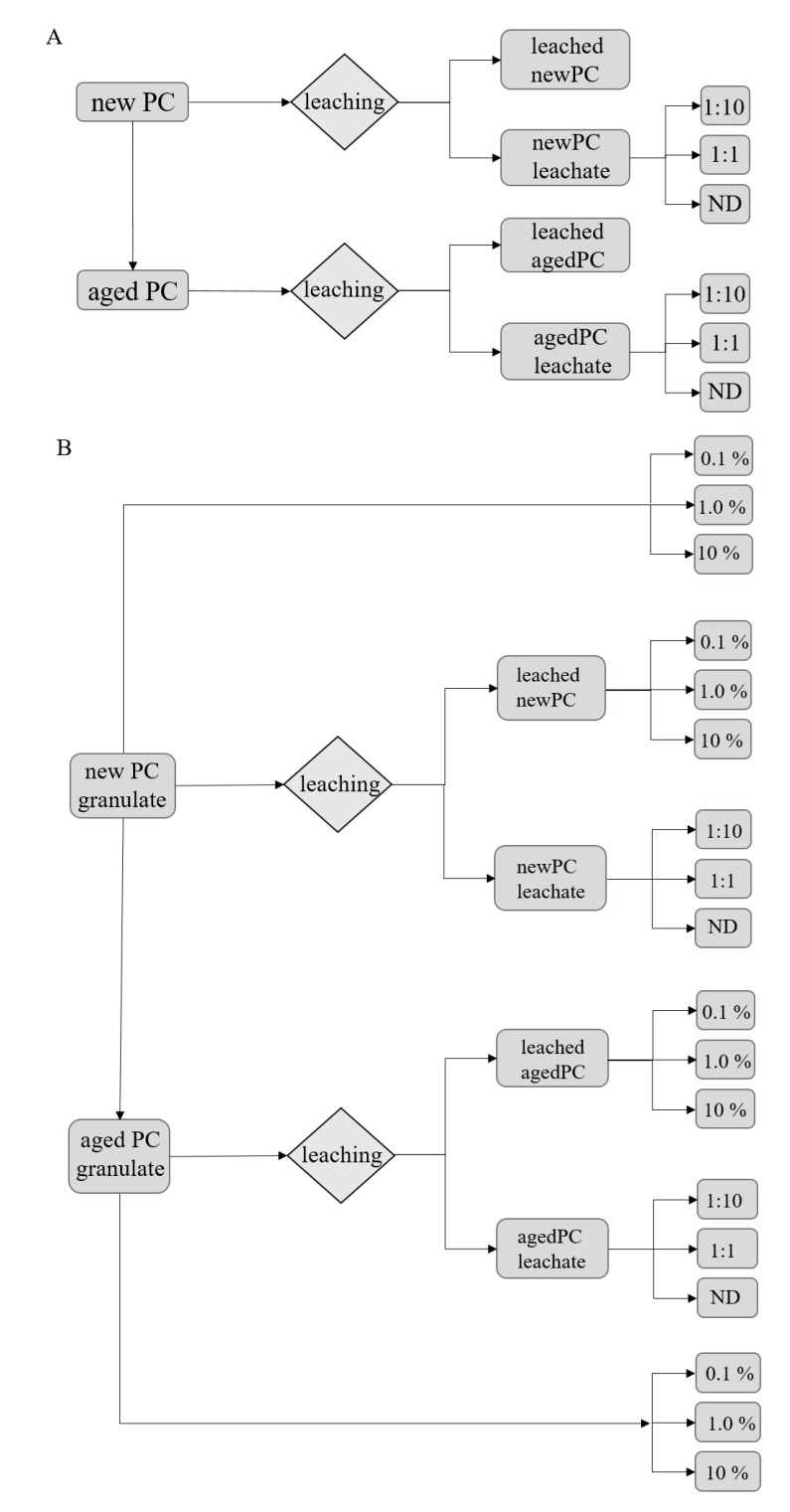
(**A**) Set up for obtaining the plastic leachates from new and artificially aged polycarbonate material and the dilutions used further one in the various exposure experiments in substrate-free exposures. (**B**) The second set up obtaining the leaches and showing the different granules treatments and percentages (w/w) used in the substrate-based experiments using *L. sativum* seeds. ND represents samples exposed to non-diluted leachate.

For the substrate-free exposures (Figure 6A), the *new* PC (*new-PC-granules*) material was used. Parts of the new PC material was artificially aged (*aged-PC-granules*) as described above. Both batches were then separately leached, as described above, to gain a leaching solution for the substrate-free experiments (*new-PC-leachate*; *aged-PC-leachate*). The leaching solution was diluted with ISO reconstitutes water 1:10, 1:1, and also non-diluted was used. For the substrate-based exposures (Figure 6B), new PC granules were used directly and mixed in the substrate in a ratio of 0.1%, 1.0%, and 10% (w/w). Parts of the new PC material was leached to gain a leaching solution (*new-PC-leachate*) and leached PC material (*new-leached-PC*). The leaching solution was diluted 1:10 and 1:1, as well as non-diluted, were used for the exposures. The leached PC material was mixed in the substrate in the ratio of 0.1%, 1.0%, and 10% (w/w). A batch of the new PC material was again artificially aged. Parts of this material was directly mixed in the substrate in the ratio of 0.1%, 1.0%, and 10% (w/w). Another part was leached to gain leached, aged PC material (*aged-leached-PC*) and the leaching solution of aged PC material (*aged-PC-leachate*). Leaching solution was diluted 1:10, 1:1, and also non-diluted were used for the exposures. The leached, aged PC material was mixed in the substrate in the ratio of 0.1%, 1.0%, and 10% (w/w).

### 3.3. Bisphenol A

Bisphenol A (BPA) is known to be an important chemical widely used as a monomer in the production of plastics. Analytical grade solid bisphenol A was obtained from Sigma-Aldrich Inc. (USA). BPA was solved in 96% ethanol to final concentrations of 10 mg/L, 50 mg/L, and 100 mg/L [24,26]. Concentrations were checked on a UPLC-MS/MS system (Acquity UPLC, Quattro Premier, Micromass, Waters GmbH, Eschborn, Germany) using the method of Vela-Soria et al. [42]. Ethanol concentration in all samples, including controls were less than 10%.

### 3.4. Control for Plastic Leachate and BPA Exposures

To investigate whether controls without and with ethanol could be grouped, ten independent substrate-free exposures with 50 seeds each of *L. sativum* was set up using ISO reconstitute water and ten independent exposures with ISO water [38] containing ethanol, to see possible effects caused by the solvent. The ethanol concentration in the medium did not exceed 0.05% (v/v). Germination was determined after seven days as well as the root length of the seedlings. Both exposures with ISO water and with water-containing ethanol (0.05%, v/v) exhibited a germination percentage of 99% ± 1%. Average root lengths of 25.1 mm ± 1.3 mm and 25.3 mm ± 1.1 mm were achieved using ISO water [38] and water containing 0.05% ethanol, respectively. As both groups were the same (*p* > 0.05), they were combined as one control for all exposures.

### 3.5. Experimental Design

All experimental setups were performed in two sets; i.e., substrate-free (Figure 6A) and substrate environment (Figure 6B) for direct comparison and a possible link to real agricultural situations.

#### 3.5.1. Substrate-Free Experiments

Seeds were sown on a Whatman filter GF/D (WH 1823-055) in glass crystallizing dishes with 5 mL of ISO reconstituted water [38] at pH 7.2 (control). Exposure solutions contained a mixture of the respective leaching solutions from new PC and aged PC material in a ration of 1:10, 1:1, and non-diluted leaching solution. Three concentrations of BPA (10.5 mg/L, 51.4 mg/L, and 103 mg/L) were used for exposures in parallel. Ten replicates with 50 seeds each were used for all exposures. The experimental set consisted of six treatments of 10 replicates (50 seeds each) and one control set with ten replicates (50 seeds each).

#### 3.5.2. Substrate Experiments

A turf-free soil-based substrate was purchased from MeinWoody (Grub am Forst, Germany) and consisted of 20% lingo fibers, 35% cocopeat washed, 10% spelt fermented, and 35% substrate compost. The substrate had a pH of 7 ± 0.5. Glass crystallizing dishes were used containing 6 g of the substrate per replicate, respectively. Exposure was done carefully mixing the respective PC material (new, aged, and leached) into the substrate until a visually homogeneous mixture was achieved in a percentage of 0.1%, 1.0%, and 10% (w/w). Before sowing the seeds, the substrate was watered with 4 mL of ISO reconstituted water [38], which had a pH 7.2; for all treatments. The different leaching solutions from *new-* and *aged-PC-granules* were directly applied onto the substrate as non-diluted or diluted with ISO reconstituted water (pH 7.2) [38] in a ratio of 1:10 and 1:1. The same three concentrations of BPA (10.5 mg/L, 51.4 mg/L, and 103 mg/L) were again applied in parallel. Each treatment had ten replicates with 50 seeds each. Every second day 500 µL of ISO reconstituted water was added for each replicate to ensure that the soil did not dry out.

During all stages of experimental set-up and analysis, attention was paid to avoid self-contamination [43].

### 3.6. Experimental End Points

The evaluation criterion for germination was the opening of the seed cover and the emergence of a 3 mm primary root. In all experiments, the following parameters were determined:

(a) ***Germination percentage*** (*GP*) is determined by calculating the level of cumulative seed germination percentage as shown in Equation (1):
  (1) $$GP=\frac{Ng}{N\;t}\;\times \;100$$

*Ng* is the number of germinated seeds, and *N t* represents the total number of seeds used in the particular batch. Germination is operationally defined as radicle emergence. The completion of germination is actually observed. The unit for *GP* is percentage (%) and measures the germination capacity [44,45,46,47,48].

(b) ***Mean total seedlings length, and radicle length*** was measured in mm after seven days of germination with a digital caliper.
(c) ***Germination rate index****(**Speed of germination; GRI**)* is expressed as the germination in terms of the total number of seeds germinating in a time interval in%/time as per Equation (2):
  (2) $$S=\;\frac{N1}{T1}+\;\frac{N2}{T2}+\ldots +\;\frac{N7}{T7}$$

*N*1, *N*2, … *N*7 are the numbers of germinated seeds observed at time (days) *T*1, *T*2, …*T*7 after sowing. No accumulated numbers but only seeds that germinated at the specific time [48,49].

### 3.7. Statistics

IBM^®^ SPSS^®^ statistics 25 (2018) was used to perform a descriptive analysis based on means of each germination index. Results are expressed as mean ± standard deviation. Data were submitted to one-way analysis of variance (ANOVA). When the overall F statistic was significant, pair-wise comparisons to controls were performed by Tukey–Kramer test [50]. The 0.05 level of significance was used.

## 4. Conclusions

Previous research has demonstrated the adverse effects of MP on animals; however, plants can be affected by MP-containing irrigation water or simply by MP in the substrate. The results of the present study showed that ageing of MP reduced the adverse effects on the germination of *L. sativum,* conceivably as harmful chemicals were leached out. Similar reduced effects were seen in the presence of substrate, where the substrate serves as a buffer and likely adsorbs the leached chemicals. Whether MP as a whole is toxic is questionable; however, leached chemicals seem to have significant adverse effects, especially in germinating seeds that soak up leachate contaminated water. In addition, it should be noted that the adverse effects might also be due to surface-bound chemicals [6] or even due to metabolic products from surface biofilms formed during the several years in the environment or a combination and should be investigated in future.

## Figures and Tables

**Table 1:** Comparison of the calculated germination rate index of *L. sativum* in substrate-free and soil exposures to leaches and granules of polycarbonate plastic particles.

	*MP Concentration*	*GRI* *Substrate-Free* *Exposures*	*GRI* *Substrate* *Exposures*
control		1.59 ± 0.06	1.71 ± 0.03
new-PC-Leachate	0.1% 1.0% 10%	1.23 ± 0.06 0.62 ± 0.03 0.22 ± 0.04	1.29 ± 0.05 1.12 ± 0.06 0.50 ± 0.06
aged-PC-Leachate	0.1% 1.0% 10%	1.50 ± 0.06 1.37 ± 0.05 1.11 ± 0.04	1.49 ± 0.06 1.27 ± 0.10 1.05 ± 0.04
BPA	10 mg/L 50 mg/L 100 mg/L	1.46 ± 0.05 1.33 ± 0.06 0.93 ± 0.05	1.63 ± 0.70 1.49 ± 0.04 1.30 ± 0.03
new-PC granules	0.1% 1.0% 10%		1.45 ± 0.05 1.20 ± 0.08 0.61 ± 0.06
aged-PC granules	0.1% 1.0% 10%		1.61 ± 0.07 1.37 ± 0.05 1.11 ± 0.03
new-PC-leached granules	0.1% 1.0% 10%		1.58 ± 0.06 1.38 ± 0.05 1.14 ± 0.02

## References

[1] Geyer R., Jambeck J.R., Law K.L. (2017). Production, use, and fate of all plastics ever made. Sci. Adv..

[2] Horton A.A., Walton A., Spurgeon D.J., Lahive E., Svendsen C. (2017). Microplastics in freshwater and terrestrial environments: Evaluating the current understanding to identify the knowledge gaps and future priorities. Sci. Total Environ..

[3] Wallace H., Jan A., Barregård L., Bignami M., Ceccatelli S., Cottrill B., Dinovi M., Edler L., Grasl-Kraupp B., Hogstrand C. (2016). Presence of microplastics and nanoplastics in food, with particular focus on seafood. EFSA Journal.

[4] Scopetani C., Chelazzi D., Cincinelli A., Esterhuizen-Londt M. (2019). Assessment of microplastic pollution: Occurrence and characterisation in Vesijarvi lake and Pikku Vesijarvi pond, Finland. Environ. Monit. Assess..

[5] Hanke G. (2013). Guidance on Monitoring of Marine Litter in European Seas.

[6] Koelmans A.A., Bergmann M., Gutow L., Klages M. (2015). Modeling the role of microplastics in bioaccumulation of organic chemicals to marine aquatic organisms. A Critical Review. Marine anthropogenic litter.

[7] Ng E.-L., Lwanga E.H., Eldridge S.M., Johnston P., Hu H.-W., Geissen V., Chen D. (2018). An overview of microplastic and nanoplastic pollution in agroecosystems. Sci. Total Environ..

[8] Nizzetto L., Futter M., Langaas S. (2016). Are agricultural soils dumps for microplastics of urban origin?. Environ. Sci. Technol..

[9] Nizzetto L., Langaas S., Futter M. (2016). Pollution: Do microplastics spill on to farm soils?. Nature.

[10] Besseling E., Quik J.T.K., Sun M., Koelmams A.A. (2016). Fate of nano- and microplastic in freshwater systems: A modeling study. Environ. Pollut..

[11] Piehl S., Leibner A., Löder M.G.J., Dris R., Bogner C., Laforsch C. (2018). Identification and quantification of macro- and microplastics on agricultural farmland. Sci. Rep..

[12] Rillig M. (2012). Microplastic in terrestrial ecosystems and the soil. Environ. Sci. Technol..

[13] Weithmann N., Möller J.N., Löder M.G.J., Piehl S., Laforsch C., Freitag R. (2018). Organic fertilizer as a vehicle for the entry of microplastic into the environment. Sci. Adv..

[14] de Souza Machado A.A., Kloas W., Zarfl C., Hempel S., Rillig M.C. (2018). Microplastics as an emerging threat to terrestrial ecosystems. Glob. Change Biol..

[15] de Souza Machado A.A., Lau C.W., Till J., Kloas W., Lehmann A., Becker R., Rillig M.C. (2018). Impacts of microplastics on the soil biophysical environment. Environ. Sci. Technol..

[16] Pflugmacher S., Huttunen J.H., von Wolff M.A., Penttinen O.P., Kim Y.J., Kim S., Mitrovic S.M., Esterhuizen-Londt M. (2020). *Enchytraeus crypticus* avoid soil spiked with microplastic. Toxics.

[17] Lithner D., Larsson A., Dave G. (2011). Environmental and health ranking and assessment of plastic polymers based on chemical composition. Sci. Total Environ..

[18] Hermabessiere L., Dehaut A., Paul-Pont I., Lacroix C., Jezequel R., Soudant P., Duflos G. (2017). Occurrence and effects of plastic additives on marine environments and organisms: A review. Chemosphere.

[19] Crompton T. (2007). Additive migration from plastics into foods. A guide for analytical chemistry.

[20] Møller L. Environmental Project no. 901, 2004. Retrieved 2010, from Evaluation of Alternatives for Compounds under Risk Assessment in the EU, Bisphenol A. http://www.miljoindflydelse.dk/common/Udgivramme/Frame.asp?http://www.miljoindflydelse.dk/udgiv/publications/2004/87-7614-181-0/html/helepubl_eng.htm.

[21] Labouriau L.G. (1983). A germinação das sementes. Organização dos Estados Americanos. Programa Regional de Desenvolvimento Científico e Tecnoloógico. Série de Biologia., Monografia.

[22] Parkpian P., Leong S.T., Laortanakul P., Juntaramitree J. (2002). An environmentally sound method for disposal of both ash and sludge wastes by mixing with soil: A case study of Bangkok plain. Environ. Monit. Assess..

[23] Staples C.A., Dome P.B., Klecka G.M., Oblock S.T., Harris L.R. (1998). A review of the environmental fate, effects, and exposures of bisphenol A. Chemosphere.

[24] Dogan M., Yumrutas O., Saygideger S.D., Korkunc M., Gulnaz O., Sokmen A. (2010). Effects of bisphenol A and tetrabromobisphenol A on chickpea roots in germination stage. Am. Eurasian, J. Agric. Environ. Sci..

[25] Dogan M., Korkung M., Yumrutas O. (2012). Effects of bisphenol A and tetrabromobisphenol A on bread and durum wheat varieties. Ekoloji.

[26] Al-Hiyasat A. (2017). The effect of bisphenol A on root development and chlorophyll a:b ratio in *Lens culinaris*. Int. J. Sci.: Basic Appl. Res..

[27] Rillig M.C., Lehmann A., de Souza Machado A.A., Yang G. (2019). Microplastic effects on plants. New Phytol..

[28] Maila M.P., Cloete T.E. (2002). Germination of *Lepidium sativum* as a method to evaluate polycyclic aromatic hydrocarbons (PAHs) removal from contaminated soil. Int. Biodeter. Biodegr..

[29] Bosker T., Bouwman L.J., Brun N.R., Behrens P., Vijver M.G. (2019). Microplastics accumulate on pores in seed capsule and delay germination and root growth of the terrestrial vascular plant *Lepidium sativum*. Chemosphere.

[30] Wan Y., Wu C.C., Xue Q., Hui X. (2019). Effects of plastic contamination on water evaporation and desiccation cracking in soil. Sci. Total Environ..

[31] Ferrara G., Loffredo E., Senesi N. (2006). Phytotoxic, clastogenic and bioaccumulation effects of the environmental endocrine disruptor bisphenol A in various crops grown hydroponically. Planta.

[32] Sun H., Wang L., Zhou Q. (2013). Effects of bisphenol A on growth and nitrogen nutrition of roots of soybean seedlings. Environ. Toxicol. Chem..

[33] Nicola S. (1998). Understanding root systems to improve seedling quality. Hort. Technol..

[34] Leskovar D.I., Stoffella P.J. (1995). Vegetable seedling root systems: Morphology, development and importance. HortScience.

[35] Zimmermann R.P., Kardos L.T. (1961). Effect of bulk density on root growth. Soil Sci..

[36] Esechie H. (1994). Interaction of salinity and temperature on the germination of sorghum. J. Agron. Crop Sci..

[37] Sommer F., Dietze V., Baum A., Sauer J., Gilge S., Maschowski C., Giere R. (2018). Tire abrasion as a major source of microplastics in the environment. Aerosol Air Qual. Res..

[38] International Organization for Standardization (ISO) (1996). Water Quality - Determination of the Acute Lethal Toxicity of Substances to a Freshwater Fish [Brachydanio Rerio Hamilton-Buchanan (Teleostei, Cyprinidae)].
ISO 7346-3: Flow-through Method.

[39] Fejdyś M., Landwijt M., Strusczyk M.H. (2011). Effects of accelerated ageing conditions on the degradation process of dyneema ^®^ polyethylene composites. Fibres Text. East Eur..

[40] Swedish Standards Institute (SIS) (2003). Characterization of Waste - Leaching - Compliance Test for Leaching of Granular Waste Materials and Sludges.

[41] Wypych G. (1999). Weathering of plastics: Testing to mirror life performance.

[42] Vela-Soria F., Ballesteros O., Zafra-Gòmez A., Ballesteros L., Navalòn A. (2014). UHPLC-MS/MS method for the determination of bisphenol A and its chlorinated derivatives, bisphenol S, parabens, and benzophenones in human urine samples. Anal. Bioanal. Chem..

[43] Scopetani C., Esterhuizen-Londt M., Chelazzi D., Cincinelli A., Setälä H., Pflugmacher S. (2020). Self-contamination from clothing in microplastics research. Ecotoxicol. Environ. Saf..

[44] Janssen J.G.M. (1973). A method of recording germination curves. Ann. Bot..

[45] Scott S.J., Jones R.A., Williams W.A. (1984). Review of data analysis for seed germination. Crop Sci..

[46] International Seed Testing Association (ISTA) (1985). International rules for seed testing. Seed Sci. Technol..

[47] International Seed Testing Association (ISTA) (2015). Chapter 5: The germination test. International Rules for Seed Testing.

[48] Throneberry G.O., Smith F.G. (1955). Relation of respiratory and enzymatic activity to corn seed viability. Plant Physiol..

[49] Wardle D.A., Ahmed M., Nicholson K.S. (1991). Allelopathic influence of nodding thistle (*Carduus nutans* L.) seeds on germination and radicle growth of pasture plants. New Zeal. J. Agr. Res..

[50] Sokal R.R., Rohlf F.J. (1997). Biometry. The Principles and Practice of Statistic in Biological Research.

